# *Arabidopsis* membrane-associated acyl-CoA-binding protein ACBP1 is involved in stem cuticle formation

**DOI:** 10.1093/jxb/eru304

**Published:** 2014-07-22

**Authors:** Yan Xue, Shi Xiao, Juyoung Kim, Shiu-Cheung Lung, Liang Chen, Julian A. Tanner, Mi Chung Suh, Mee-Len Chye

**Affiliations:** ^1^School of Biological Sciences, The University of Hong Kong, Pokfulam Road, Hong Kong, China; ^2^State Key Laboratory of Biocontrol and Guangdong Key Laboratory of Plant Resources, School of Life Sciences, Sun Yat-sen University, Guangzhou 510275, China; ^3^Department of Bioenergy Science and Technology, Chonnam National University, Gwangju 500-757, Korea; ^4^Department of Biochemistry, The University of Hong Kong, Pokfulam, Hong Kong, China

**Keywords:** Acyl-CoA-binding protein, *Arabidopsis thaliana*, cuticle, cuticular wax, cutin, very-long-chain acyl-CoAs.

## Abstract

The membrane-anchored *Arabidopsis thaliana* ACYL-COA-BINDING PROTEIN1 (AtACBP1) plays important roles in embryogenesis and abiotic stress responses, and interacts with long-chain (LC) acyl-CoA esters. Here, AtACBP1 function in stem cuticle formation was investigated. Transgenic *Arabidopsis* transformed with an *AtACBP1pro::GUS* construct revealed β-glucuronidase (GUS) expression on the stem (but not leaf) surface, suggesting a specific role in stem cuticle formation. Isothermal titration calorimetry results revealed that (His)_6_-tagged recombinant AtACBP1 interacts with LC acyl-CoA esters (18:1-, 18:2-, and 18:3-CoAs) and very-long-chain (VLC) acyl-CoA esters (24:0-, 25:0-, and 26:0-CoAs). VLC fatty acids have been previously demonstrated to act as precursors in wax biosynthesis. Gas chromatography (GC)–flame ionization detector (FID) and GC–mass spectrometry (MS) analyses revealed that an *acbp1* mutant showed a reduction in stem and leaf cuticular wax and stem cutin monomer composition in comparison with the wild type (Col-0). Consequently, the *acbp1* mutant showed fewer wax crystals on the stem surface in scanning electron microscopy and an irregular stem cuticle layer in transmission electron microscopy in comparison with the wild type. Also, the mutant stems consistently showed a decline in expression of cuticular wax and cutin biosynthetic genes in comparison with the wild type, and the mutant leaves were more susceptible to infection by the necrotrophic pathogen *Botrytis cinerea*. Taken together, these findings suggest that AtACBP1 participates in *Arabidopsis* stem cuticle formation by trafficking VLC acyl-CoAs.

## Summary

The binding of recombinant AtACBP1 to very-long-chain acyl-CoA esters is related to AtACBP1 function in *Arabidopsis* stem cuticle metabolism. Loss-of-function mutation adversely affected stem cuticle composition and structure.

## Introduction

The *Arabidopsis* cuticle is a lipophilic layer that consists of cutin and wax (Nawrath, 2002; Kunst and Samuels, 2009; Lee and Suh, 2013). Cutin is a polymer derived from hydroxy and epoxy-hydroxy C16 and C18 fatty acids (Nawrath, 2006). Cutin biosynthesis consists of sequential reactions including the activation of acyl chains to coenzyme A by long-chain acyl-CoA synthetase (LACS), hydroxylation and epoxidation catalysed by the cytochrome P450 family, and esterification to glycerol-3-phosphate by glycerol-3-phosphate acyltransferases (GPATs) (Li-Beisson *et al.*, 2013). Waxes, including epicuticular waxes that cover the cuticle membrane and intracuticular waxes embedded in the cuticle membrane, are complex mixtures of alcohols, alkanes, aldehydes, ketones, and esters derived from long-chain fatty acids (Jenks *et al.*, 2002). In plant epidermal cells, saturated very-long-chain fatty acids (VLCFAs), comprising acyl chains exceeding 20 carbons (>C20) generated by the extension of C16 and C18 fatty acids in the endoplasmic reticulum (ER), form precursors for the synthesis of aliphatic components of cuticular waxes (Kunst and Samuels, 2003).

Acyl-CoA-binding proteins (ACBPs) constitute a family of eukaryotic proteins that show conservation in an acyl-CoA-binding (ACB) domain with ability to bind long-chain acyl-CoA esters (Knudsen *et al.*, 2000; Xiao and Chye, 2009, 2011a; Fan *et al.*, 2010; Yurchenko and Weselake, 2011). In the model plants *Arabidopsis thaliana* and *Oryza sativa*, six genes designated as *AtACBP1*–*AtACBP6* and *OsACBP1*–*OsACBP6*, respectively, encode ACBPs that bind acyl-CoA esters and phospholipids with varying affinities (Engeseth *et al.*, 1996; Chye, 1998; Chye *et al.*, 2000; Leung *et al*., 2004, 2006; Chen *et al.*, 2008; Gao *et al.*, 2009; Xiao *et al.*, 2009; Meng *et al.*, 2011, 2014; Du *et al.*, 2013*b*). Variation in subcellular localization has also been observed (Chye *et al.*, 1999; Li and Chye, 2003; Leung *et al.*, 2006; Xiao *et al.*, 2008*b*; Xiao and Chye, 2009; Meng *et al.*, 2014). Thus, given the differences in subcellular localization and substrate preference, it appears that some *Arabidopsis* ACBPs perform distinct cellular functions *in vivo* while others with similar subcellular localization and binding affinities for acyl-CoA esters may share overlapping roles (Chye *et al*., 1999, 2000; Li and Chye, 2003; Leung *et al.*, 2006; Xiao *et al.*, 2008*b*; Xiao and Chye, 2009, 2011a). For example, AtACBP1 and AtACBP2 both show functions in embryogenesis (Chen *et al.*, 2010) and seedling development (Du *et al*., 2013*a*, *b*), while AtACBP3 promotes starvation-induced leaf senescence, age-dependent leaf senescence (Xiao *et al.*, 2010), and plant defence against *Pseudomonas syringae* (Xiao and Chye, 2011*b*; Zheng *et al.*, 2012). AtACBPs are also associated with heavy metal/oxidative (Xiao *et al.*, 2008*a*; Gao *et al*., 2009, 2010), freezing (Chen *et al.*, 2008; Du *et al.*, 2010; Liao *et al.*, 2014), and drought (Du *et al.*, 2013*a*) stresses. AtACBP1 (Du *et al.*, 2013*b*) and AtACBP2 (Gao *et al*., 2009, 2010) both mediate protein–protein interactions by binding transcription factors and stress-responsive partners.

AtACBP1 was subcellularly localized to the ER and the plasma membrane (PM) (Li and Chye, 2003), and immunoelectron microscopy using anti-AtACBP1 antibody revealed that the AtACBP1 protein accumulates in developing embryos (Chye *et al.*, 1999). The roles of AtACBP1 in embryo development were confirmed by phenotypic and biochemical studies using the *acbp1* T-DNA insertional mutant (Chen *et al.*, 2010). Alterations in membrane lipid composition and acyl-CoA content in the *acbp1* siliques were observed. In addition, the observation of arrest of early embryo development in the *acbp1acbp2* double mutant suggested that AtACBP1 and AtACBP2 are essential during early embryogenesis in *Arabidopsis* (Chen *et al.*, 2010), most probably in lipid transfer because (His)_6_-tagged recombinant AtACBP1 (rACBP1) and AtACBP2 bind acyl-CoA esters and both demonstrated preference for unsaturated over saturated long-chain acyl-CoA esters (Chye, 1998; Chye *et al.*, 2000; Leung *et al.*, 2006; Gao *et al.*, 2009).

AtACBP1 has been observed to accumulate in the outer integument cells of the developing seed coat and has been previously proposed to be involved in the biosynthesis of cutin and cuticular waxes (Chye *et al.*, 1999). A membrane-associated ACBP from *Agave americana* showing 62% amino acid identity to AtACBP1, AaACBP1, was enriched in the epidermis of mature leaves (Guerrero *et al.*, 2006). Besides the presence of the conserved ACB domain, AaACBP1 contains ankyrin repeats which potentially mediate protein–protein interactions (Michaely and Bennett, 1992) similarly to AtACBP1 (Xiao and Chye, 2011*a*). The expression of AaACBP1 in the epidermal cells (Guerrero *et al.*, 2006) and observation of AtACBP1 localization at the endomembranes (Chye *et al.*, 1999) support the feasibility of these ACBPs as candidates involved in the biosynthesis of cuticular lipids (Chye *et al*, 1999; Kunst and Samuels, 2003; Xiao and Chye, 2011*a*; Li-Beisson *et al.*, 2013). Interestingly, in mice, ACBP has been reported to be essential in the formation of an epidermal barrier (Bloksgaard *et al.*, 2012), while Xia *et al.* (2012) demonstrated that AtACBP3, AtACBP4, and AtACBP6 function in cuticle formation in *Arabidopsis*. To address the role of AtACBP1 in cuticle formation, it was first demonstrated that (His)_6_-tagged rACBP1 can bind very-long-chain (VLC) acyl-CoA esters *in vitro*. Subsequently, investigations on the *acbp1* T-DNA insertional mutant showed that it displayed reduction in cuticular wax and cutin monomer composition in *Arabidopsis* stems, suggesting that AtACBP1 functions in stem cuticle formation.

## Materials and methods

### Plant materials and growth conditions

Seeds of wild-type *A. thaliana* (ecotype Col-0), the *acbp1* mutant (SAIL-653-B06, ecotype Col-0; Xiao *et al.*, 2008*a*), and *acbp1-COM* (Xiao *et al.*, 2008*a*) were surface-sterilized, cold-stratified, and germinated on Murashige and Skoog medium (MS medium) (Murashige and Skoog, 1962) supplemented with 2% sucrose for 10 d under cycles of 8h dark (21 °C) and 16h light (23 °C). Plants were transferred into soil and were grown in a growth chamber under a 16h light/8h dark cycle. Stems were harvested from 6-week-old plants for gas chromatography (GC) analysis following Lee *et al.* (2009*b*).

### Expression and purification of rACBP1

(His)_6_-AtACBP1 recombinant protein was expressed in the soluble fraction of *Escherichia coli* BL21(DE3), and was purified through Ni-NTA agarose (Qiagen, Valencia, CA, USA) affinity columns as previously described (Chye, 1998).

### Isothermal titration calorimetry (ITC) measurements

ITC experiments were performed using an isothermal titration calorimeter (MicroCal iTC_200_ system) from MicroCal Inc. (USA). Long-chain and VLC acyl-CoA esters used in this study were purchased from Avanti Polar Lipids (http://www.avantilipids.com/). The acyl-CoA concentration (250 μM) in the titration syringe was 25-fold higher than the protein concentration (10 μM) in the cell. Acyl-CoA solutions and rACBP1 protein were degassed under vacuum and stirred immediately before use. The experiments were performed at 30 °C, and injections were initiated after equilibration to baseline stability. Each injection was made up to a volume of 1.5 μl and lasted 10 s, with an interval of 240 s between injections. The syringe was rotated at 1000rpm during the assay to ensure immediate mixing. Raw data were integrated, corrected for non-specific heat, and analysed using the ORIGIN software supplied with the instrument by the General Electric Company. The dissociation constant (*K*_D_) was calculated by non-linear regression fitting the isotherm.

### β-Glucuronidase (GUS) histochemical assays

GUS histochemical assays were carried out on *AtACBP1pro::GUS Arabidopsis* transformed with construct pAT352 according to Du *et al.* (2013*b*). The standard 5-bromo-4-chloro-3-indolyl-β-d-glucuronide (X-Gluc) solution (100mM sodium phosphate buffer, pH 7.0, 0.1% Triton X-100, 1mg ml^–1^ X-Gluc) with the addition of 2mM potassium ferricyanide and 2mM potassium ferrocyanide was used. Leaves and stems from 4-week-old *AtACBP1pro::GUS* transgenic *Arabidopsis* were vacuum-infiltrated in X-Gluc solution for 30min and kept at 37 °C until a blue colour developed. Samples were destained in 70% ethanol and photographed. The controls in the GUS assays were samples from Col-0 and transgenic *Arabidopsis* transformed with vector pBI101.3, and they were not stained blue during the same incubation period. The GUS-stained stems and leaves were embedded in Paraplast for sectioning according to Sin *et al.* (2006).

### Quantitative real-time polymerase chain reactions (qRT-PCRs)

Total RNA was isolated from stems of five 5-week-old *Arabidopsis* plants using the RNeasy Plant Mini Kit following the protocol provided by QIAGEN. RNA (3.5 μg) was reverse-transcribed into cDNA using the SuperScript First-Strand Synthesis System (Invitrogen). PCR was conducted on a StepOne Plus Real-time PCR system using SYBR Green Mix (Applied Biosystems) in the following steps: 10min at 95 °C followed by 40 cycles of 95 °C (15 s) and 56 °C (1min). For each reaction, three experimental replicates were performed with gene-specific primers, and *Arabidopsis ACTIN2* was used as an internal control (Supplementary Table S1 available at *JXB* online). The relative expression of the targeted gene was normalized using the *ACTIN*2 control as described (Xiao *et al.*, 2011*b*).

### Scanning electron microscopy (SEM)

Stems of the first internodes above the rosette from 6-week-old wild-type *Arabidopsis* and the *acbp1* mutant were used. Samples were treated with 1% osmium tetroxide (OsO_4_) for 24h and then air-dried for 3 d, followed by mounting onto standard aluminium stubs and sputter coating with gold particles using six 30-s bursts according to Chen *et al.* (2003). The coated samples were viewed with a Hitachi S3400 scanning electron microscope.

### Transmission electron microscopy (TEM)

The ultrastructure of 6-week-old stems from wild-type *Arabidopsis* and the *acbp1* mutant were prepared for TEM following Sieber *et al.* (2000) with some modifications. Samples were fixed using 2.5% glutaraldehyde in cacodylate buffer (0.1M sodium cacodylate-HCl buffer, pH 7.4) for 4h at 4 °C, followed by post-fixation treatment with 1% osmium tetroxide in cacodylate buffer for 4h at 4 °C. After gradient dehydration with ethanol, samples were infiltrated overnight in an epoxy resin/propylene oxide 1:1 mixture. This was followed by infiltration overnight in epoxy resin. Samples were subsequently embedded in epoxy resin and polymerized overnight at 60 °C. Ultrathin (60nm) sections were prepared and stained with 2% uranyl acetate and lead citrate (Li *et al.*, 2008), and subsequently examined using a Phillips CM100 transmission electron microscope.

### Wax analysis

Cuticular waxes were extracted by immersing two stem segments (each 10-cm in length) in 5ml of chloroform for 30 s. The internal standards used were C28 alkane (*n*-octacosane), C22 fatty acid (docosanoic acid), and C23:0 fatty alcohol (1-tricosanol). The solvent was then removed by heating (40 °C) under a mild stream of nitrogen. Derivatization was performed by adding 100 μl of pyridine and 100 μl of bis *N*,*N*-(trimethylsilyl) trifluoroacetamide (Sigma) to the dried extract and incubating for 30min at 90 °C. The qualitative composition was then evaluated by capillary GC–mass spectrometry (GC-MS; GCMS-QP2010; Shimadzu; column, 60 m HP-5, 0.32mm id, film thickness=0.25 μm; Agilent) using a helium carrier gas inlet pressure of 1.0ml min^–1^ and a mass spectrometric detector (GCMS-QP2010; Shimadzu). GC-MS conditions were as follows: injection at 220 °C, maintenance of the temperature at 220 °C for 4.5min, followed by an increase to 290 °C at a rate of 3 °C min^–1^. The temperature was then maintained at 290 °C for 10min, after which it was raised to 300 °C at a rate of 2 °C min^–1^ and held for 10min (Lee *et al.*, 2009*b*). Analysis of quantitative wax materials was performed using a capillary GC program with a flame ionization detector (FID) using the same conditions as in GC-MS. Compounds were quantified relative to the corresponding internal standards by integrating the peak areas.

### Cutin analysis

Five- to six 6-week-old primary stems of *Arabidopsis* were used for cutin analysis according to Lee *et al.* (2009*a*). The internal standards used were C17:0 methyl ester (methyl heptadecanoate) and C15:0 cycloketone (ω-pentadecalactone) (Sigma). Polyesters from dried solvent-extracted residues of stems (wax-free) were depolymerized by hydrogenolysis with methanolysis using sodium methoxide. The products recovered after hydrogenolysis were dried and derivatized as mentioned above and separated and quantiﬁed by GC-MS. The GC-MS protocol was as follows: injection at 110 °C, elevation by 2.5 °C min^–1^ to 300 °C, and holding for 3min at 300 °C. The mass-to-charge ratios (*m/z*) used to diagnose the cutin compounds are shown in Supplementary Table S2 at *JXB* online.

### Inoculation of plants with *Botrytis cinerea*

The necrotrophic fungus *B. cinerea* was maintained on a potato dextrose agar plate (BD Difco) at room temperature. Collection of conidia and plant infection assays (*Botrytis* suspension concentration of 2×10^5^ spores ml^–1^) were carried out as previously described (Li *et al*., 2008; Xiao and Chye, 2011*b*). Photographs were taken at 0 and 6 days after infection (DAI).

### Accession numbers

Sequence data from this article can be found in the Arabidopsis Genome Initiative database under the following accession numbers: At5g53470 (*ACBP1*), At3g18780 (*ACTIN2*), At2g47240 (*CER8*), At1g67730 (*KCR1*), At3g55360 (*ECR*), At1g68530 (*CUT1/KCS6*), At1g49430 (*LACS2*), At4g00360 (*CYP86A2*), At1g01600 (*CYP86A4*), At4g00400 (*GPAT8*), At2g19450 (*DGAT1*), At3g51520 (*DGAT2*), At1g48300 (*DGAT3*), At5g13640 (*PDAT1*), At1g20440 (*COR47*), At5g52310 (*LTI78*), At4g25490 (*CBF1*), and At3g26744 (*ICE1*).

## Results

### Recombinant AtACBP1 binds VLC acyl-CoA esters (C24:0-, C25:0-, and C26:0-CoA) *in vitro*

**Fig. 1.**
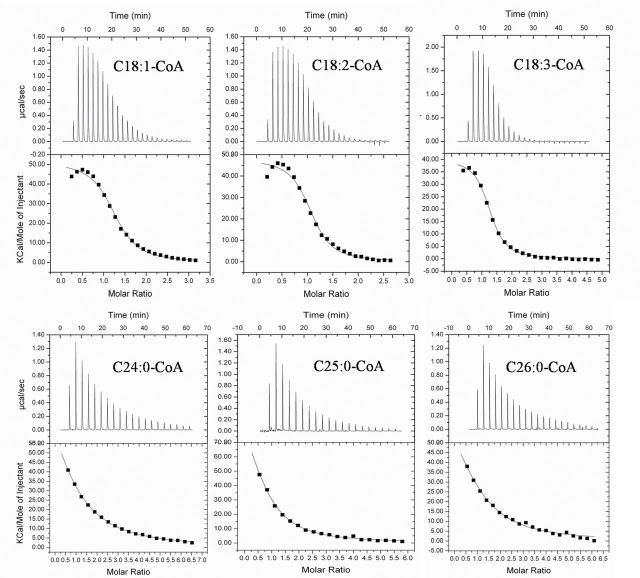
Binding isotherms of recombinant AtACBP1 titrated with C18:1-, C18:2-, C18:3-, C24:0-, C25:0-, and C26:0-CoA esters at 30 °C using isothermal titration calorimetry. Top panels show raw data of 300 μl of 10 μM recombinant AtACBP1 titrated with 20 injections of 1.5 μl of 250 μM acyl-CoA ester solution. Bottom panels show the integrated area of each injection and the plotted graph. Parameters of the dissociation constant (*K*_D_) are given in Table 1.

(His)_6_-tagged rACBP1 was shown to bind long-chain acyl-CoA esters (C18:1-, C18:2-, and C18:3-CoA) in Lipidex assays (Leung *et al.*, 2006) and gel-binding assays (Chye, 1998). As rACBPs have not been reported to bind VLC acyl-CoA esters, rACBP1 was tested using commercially available VLC acyl-CoA esters (C24:0-, C25:0-, and C26:0-CoA) by ITC to determine the *K*_D_ values. As controls, long-chain acyl-CoA esters (C18:1-, C18:2-, and C18:3-CoA) were included. Analysis of calorimetric data by the ORIGIN software (General Electric Company, USA) indicated that the binding isotherms fitted well with a model of a single binding site (Fig. 1). Consistent with the results from Lipidex assays (Leung *et al.*, 2006) and gel-binding assays (Chye, 1998), rACBP1 interacted with long-chain acyl-CoA esters including C18:1-, C18:2-, and C18:3-CoAs with high affinities in ITC measurements (Fig. 1; Table 1). Furthermore, rACBP1 was also shown to bind to VLC acyl-CoA esters (C24:0-, C25:0-, and C26:0-CoA), although with lower affinities (Fig. 1; Table 1). The affinities as reflected by the *K*_D_ values of rACBP1 for VLC acyl-CoA esters supported their participation in faty acid elongation during the biosynthesis of VLCFAs.

**Table 1.** The dissociation constants (*K*_D_) of recombinant AtACBP1 (rACBP1) binding to acyl-CoA esters of different acyl chain lengthsThe values are means ±SD (*n*=3).

Acyl-CoA esters	*K*_D_ (μM)
C18:1	0.76±0.15
C18:2	0.83±0.04
C18:3	0.44±0.01
C24:0	2.14±0.13
C25:0	1.69±0.11
C26:0	1.94±0.12

### *AtACBP1* is expressed in stem epidermis

**Fig. 2.**
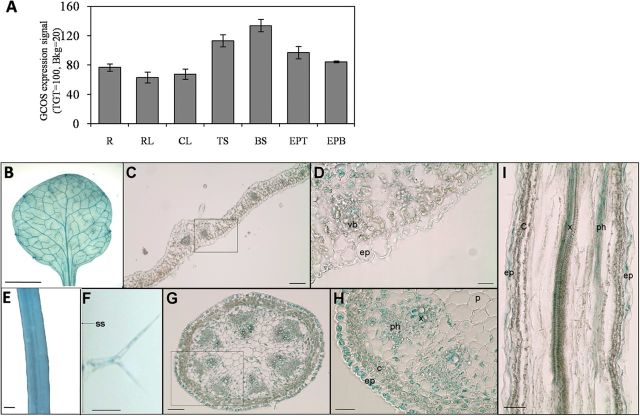
Microarray data and GUS staining for the expression of *AtACBP1*. (A) Expression pattern of *AtACBP1* in vegetative tissues of *Arabidopsis* including roots (R), rosette leaves (RL), cauline leaves (CL), top of stems (TS), bottom of stems (BS), and epidermal peels from the top (EPT) and bottom (EPB) of stems. The data were retrieved from the microarray database e-FP Browser (Winter *et al.*, 2007; http://www.bar.utoronto.ca/efp/cgi-bin/efpWeb.cgi). GCOS, gene chip operating software, the method used by Affymetrix MAS5.0 to normalize the microarray data. TGT (target) and Bkg (background) are parameters used in the normalization. (B–I) GUS expression of *AtACBP1pro::GUS* in 4-week-old transgenic *Arabidopsis*. Three independent transgenic lines were tested by staining with 1mM 5-bromo-4-chloro-3-indolyl-β-d-glucuronide with consistent results. (B) Rosette leaf; (C, D) cross-section of a rosette leaf; (E) stem; (F) trichome on the side stem; (G, H) cross-section of a stem; (I) longitudinal section of a stem. Scale bar=10mm (B); 100 μm (C); 25 μm (D, H); 2mm (E); 10 μm (F); 50 μm (G,I). vb, vascular bundle; ep, epidermis; ss, stem surface; c, cortex; ph, phloem; x, xylem; p, pith.

*AtACBP1* mRNA has been previously reported to be expressed in all plant organs (Chye, 1998; Chen *et al.*, 2010). The microarray database e-FP Browser (Winter *et al.*, 2007; http://www.bar.utoronto.ca/efp/cgi-bin/efpWeb.cgi) revealed that *AtACBP1* was highly expressed in the top and bottom of stems, in comparison with roots and rosette and cauline leaves, and that this expression localized in the epidermal peels (Fig. 2A).

The expression of *AtACBP1* in leaves and stems was investigated in transgenic *Arabidopsis* lines expressing *AtACBP1pro::GUS* (Fig. 2B–I). *AtACBP1pro::GUS* was expressed in the leaf vasculature (Fig. 2B–D) and on the stem surface (Fig. 2E) including the trichomes (Fig. 2F). The cross- and longitudinal-sections of the stem showed *AtACBP1pro::GUS* expression in the epidermis, the cortex, and the vascular bundles (Fig. 2G–I). Stem and leaf sections from control *Arabidopsis* transformed with pBI101.3 were not stained.

### The *acbp1* mutant shows defects in epicuticular wax crystallization and cuticle membrane structure

**Fig. 3.**
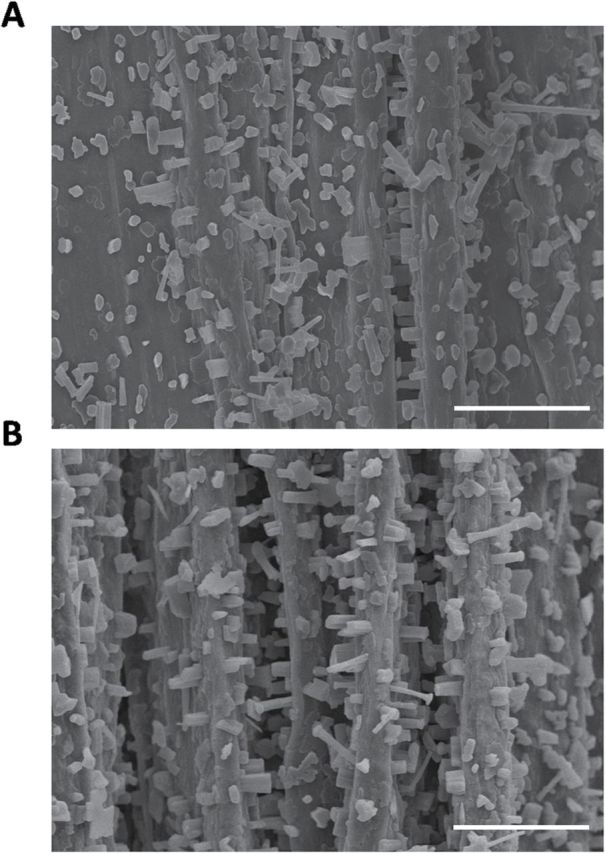
Scanning electron microscopy (SEM) analysis of epicuticular wax crystal patterns on stem surfaces of *acbp1* (A) and Col-0 (B). Scale bars=15 μm.

When SEM was used to investigate wax crystallization patterns on the stem surface of the *acbp1* mutant, the occurrence of epicuticular wax crystals was significantly reduced (Fig. 3A) in comparison with the wild type (Fig. 3B). Upright rod-, tube-, and umbrella-shaped wax crystals were arrayed in an orderly manner on the wild type, but not the *acbp1* mutant stem. In contrast, the mutant had fewer crystals (Fig. 3).

**Fig. 4.**
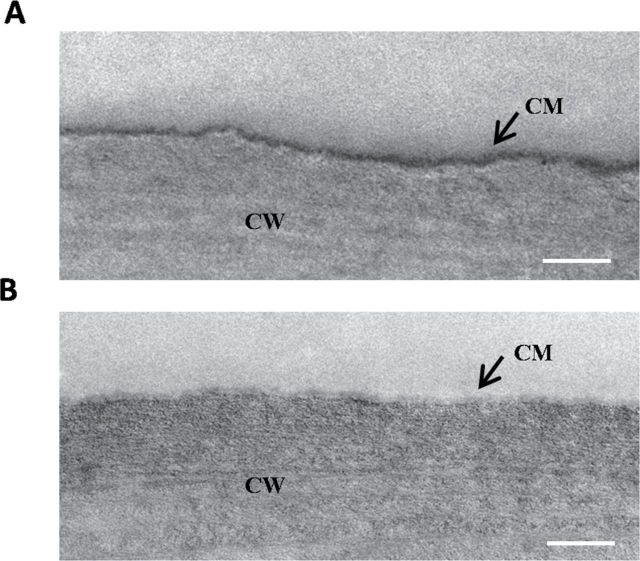
Transmission electron microscopy (TEM) of the cuticle membrane from stem epidermal cells of Col-0 (A) and the *acbp1* mutant (B). Scale bars=0.5 μm. CM, cuticle membrane; CW, cell wall.

When TEM was used to examine the fine structural changes of the stem cuticle, the cuticle membrane was intact in Col-0 (Fig. 4A) but not in the *acbp1* mutant (Fig. 4B). Instead, in the mutant, a ruptured and discontinuous cuticle membrane was observed (Fig. 4B). Absence of expression of *AtACBP1* culminated in an aberrant cuticle membrane, suggesting that AtACBP1 is involved in stem cuticle formation.

### The *acbp1* mutant shows reduction in stem cuticular wax constituents and cutin monomers and down-regulation of cuticular biosynthetic genes

To evaluate further the roles of AtACBP1 in cuticle formation, GC-FID and GC-MS were employed to determine the amount and composition of cuticular waxes from stems of the wild type (Col-0), the *acbp1* mutant, and the *acbp1*-complemented (*acbp1*-*COM*; Xiao *et al.*, 2008*a*) lines.

**Fig. 5.**
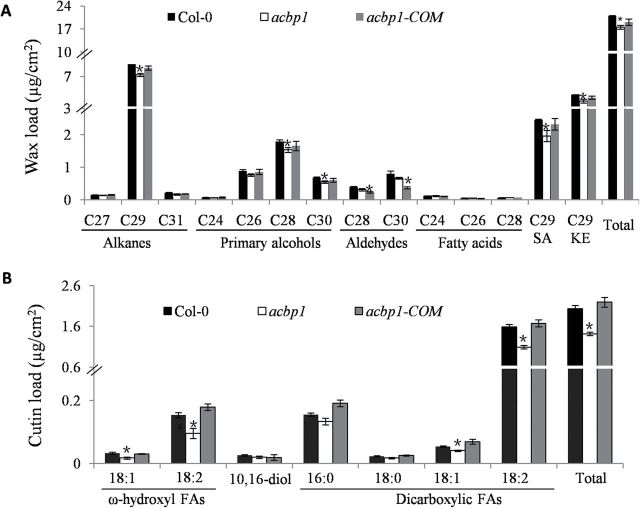
Cuticular wax (A) and cutin monomer (B) composition and amount in stems of Col-0, *acbp1*, and *acbp1-COM* plants. Six-week-old stems were used in wax and cutin analysis by GC-FID and GC-MS. SA, secondary alcohols; KE, ketones; FA, fatty acid; 10,16-diol, C16-10,16-dihydroxyl fatty acids. Asterisks denote signiﬁcant differences from the wild type (*P<0.05). Values are means ±SE (*n*=3).

**Fig. 6.**
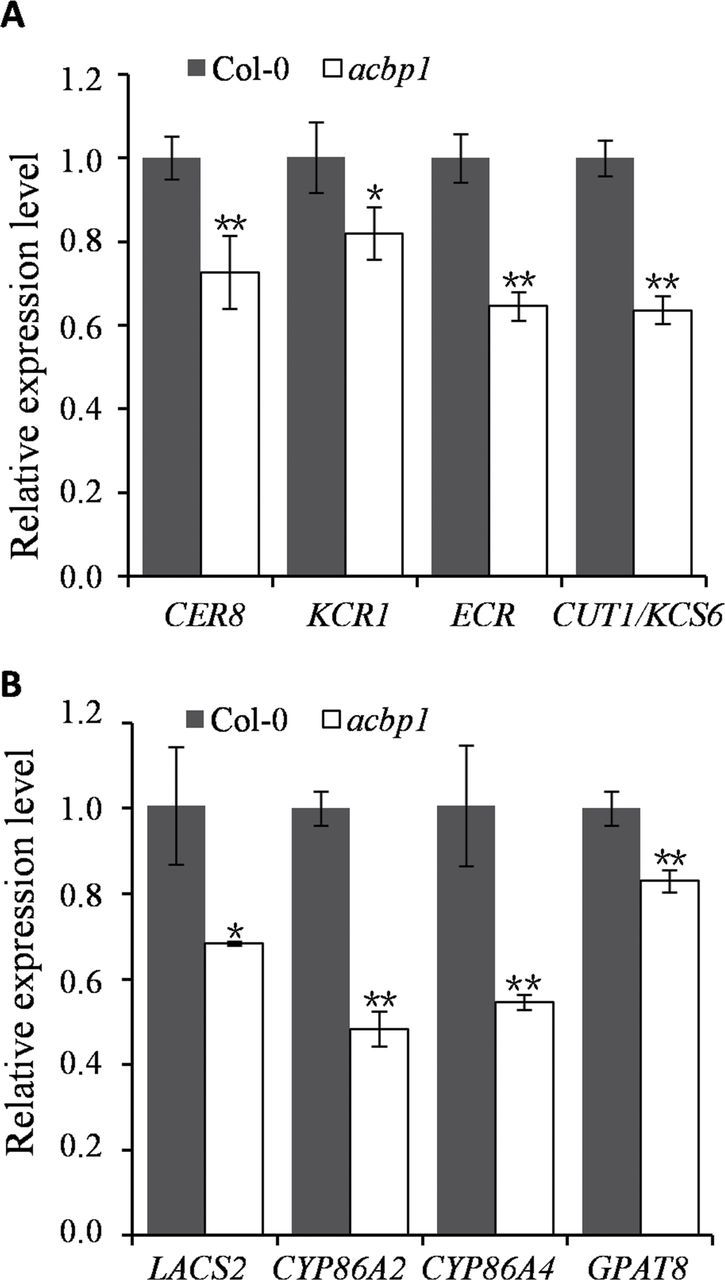
qRT-PCR analysis of wax (A) and cutin (B) biosynthetic genes in stems of the *acbp1* mutant and Col-0. Expression of *CER8*, *KCR1*, *ECR*, *CUT1/KCS6*, *LACS2*, *CYP86A2*, *CYP86A4*, and *GPAT8* decreased in stems of *acbp1* in comparison with the wild type (Col-0). Asterisks denote signiﬁcant differences from the wild type (*P<0.05; ** *P*<0.01). Values are means ±SE (*n*=3).

In stems, a 16% reduction of total wax was observed in *acbp1* in comparison with the wild type (Fig. 5A). In particular, the levels of C29 alkane, C28 and C30 primary alcohols, and C29 secondary alcohol and ketone were significantly reduced in the *acbp1* mutant (Fig. 5A). Their percentage reductions were 16% (C29 alkane), 15% (C28 primary alcohol), 21% (C30 primary alcohol), 21% (C29 secondary alcohol), and 16% (C29 ketone). The normal amounts of total wax and various wax species were recovered in stems of *acbp1-COM* (Fig. 5A), confirming that the decrease in stem cuticular wax in the *acbp1* mutant resulted from knockout of *AtACBP1* expression. Subsequently, when the expression of wax biosynthetic genes was determined by qRT-PCR analysis (Fig. 6A), *CER8*, *KCR1*, *ECR*, and *CUT1/KCS6* significantly decreased in the *acbp1* stem in comparison with the wild type (Fig. 6A), and these decreases in gene expression were recovered in the *acbp1*-*COM* plants (Supplementary Fig. S1A at *JXB* online).

GC-MS was also used to determine cutin monomer composition and amount in the stems of the *acbp1* mutant, *acbp1*-*COM*, and wild type (Fig. 5B). The amounts of total cutin monomer were altered in stems of the *acbp1* mutant (Fig. 5B) in comparison with the wild type. Levels of C18:1 and C18:2 ω-hydroxyl fatty acids, as well as C18:1 and C18:2 dicarboxylic fatty acids, were significantly reduced in stems of the *acbp1* mutant (Fig. 5B). Their percentage reductions were 45% (C18:1 ω-hydroxyl fatty acid), 38% (C18:2 ω-hydroxyl fatty acid), 24% (C18:1 dicarboxylic fatty acid), and 31% (C18:2 dicarboxylic fatty acid). The chemical change in stems of the *acbp1* mutant was recovered in the *acbp1-COM* line (Fig. 5B), confirming that reduction in the amounts of stem cutin monomer in the mutant resulted from knockout of *AtACBP1* expression. Subsequently, on qRT-PCR, expression of some genes involved in cutin synthesis (*LACS2*, *CYP86A2*, *CYP86A4*, and *GPAT8*) showed a significant decrease in the stems of *acbp1* in comparison with wild-type *Arabidopsis* (Fig. 6B), which could be recovered in *acbp1*-*COM* plants (Supplementary Fig. S1B at *JXB* online). However, the expression of triacylglycerol biosynthetic genes (*DGAT1*, *DGAT2*, *DGAT3*, and *PADT1*) (Supplementary Fig. S1C) and cold-related genes (*COR47*, *LTI78*, *CBF1*, and *ICE1*) (Supplementary Fig. S1D), which are not implicated in cuticle formation, was not affected in the *acbp1* mutant and *acbp1*-*COM* plants in comparison with the wild type.

### Seedlings of *acbp1* are more susceptible to *Botrytis cinerea* infection

**Fig. 7.**
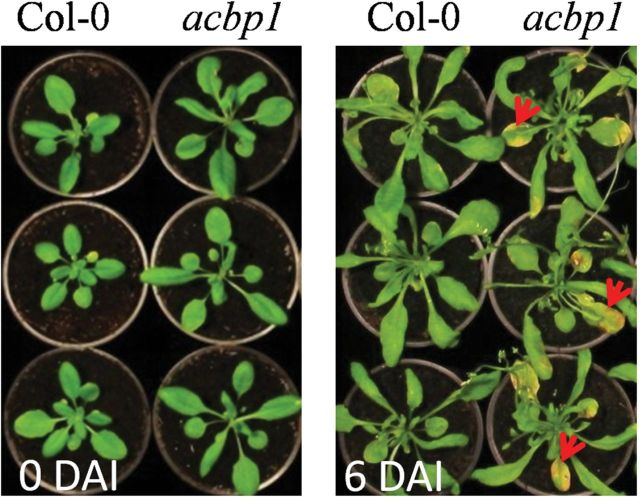
Response of *acbp1* to *Botrytis cinerea* infection. Three-week-old wild-type (Col-0) and *acbp1* plants were sprayed with *B. cinerea* (2×10^5^ spores ml^–1^). Photographs were taken at 0 and 6 days after inoculation (DAI). The experiments were repeated twice with consistent results. Arrows indicate chlorosis and necrosis of leaves.

To examine whether a reduction of wax and cutin in the *acbp1* mutant confers an altered response to the necrotrophic fungal pathogen *B. cinerea*, 3-week-old seedlings of the *acbp1* mutant and wild type were inoculated with *Botrytis* spores. As shown in Fig. 7, the *acbp1* mutant seedlings displayed enhanced susceptibility after spraying with *Botrytis* suspension. At 6 DAI, chlorosis and necrosis were observed in the *acbp1* mutant, but not in the wild type (Fig. 7). Measurement of leaf wax and cutin from the *acbp1* mutant in comparison with the wild type revealed a significant decrease in wax but not in cutin (Supplementary Fig. S2 at *JXB* online). In particular, C31 and C33 alkanes, C28 fatty acids, and total wax load significantly declined (Supplementary Fig. S2A). This is not surprising because *AtACBP1pro::GUS* was expressed more in stem epidermis than in leaf epidermis (Fig. 2). These results suggest that a reduction in wax content in the *acbp1* mutant could have caused greater susceptibility to *Botrytis* infection.

## Discussion

In plants, fatty acids are synthesized in the plastids by the addition of two-carbon units to a growing acyl chain facilitated by the acyl carrier protein (ACP) during *de novo* fatty acid synthesis (Ohlrogge and Browse, 1995). Subsequently, 16:0-ACP and 18:0-ACP are exported to the ER for the biosynthesis of other lipids including cutin, suberin, and cuticular waxes (Post-Beittenmiller, 1996; Jenks *et al.*, 2002; Nawrath, 2002). Cutin and wax are synthesized exclusively in the epidermis (Nawrath, 2002; Suh *et al.*, 2005; Samuels *et al.*, 2008). *AtACBP1pro::GUS* is expressed in the embryos, lateral root primordia, vascular bundles, stigmas, and ovaries (Du *et al.*, 2013*b*). In this study, transgenic *Arabidopsis* expressing *AtACBP1pro::GUS* showed strong GUS expression in stem epidermis, in agreement with the corresponding expression analysis of *KCS20* and *KCS2/DAISY* genes involved in VLCFA elongation (Lee *et al.*, 2009*b*). It is noteworthy that the GUS stain was not detected in leaf epidermis (Fig. 2D), suggesting a putative function for AtACBP1 in stem cuticle formation.

**Fig. 8.**
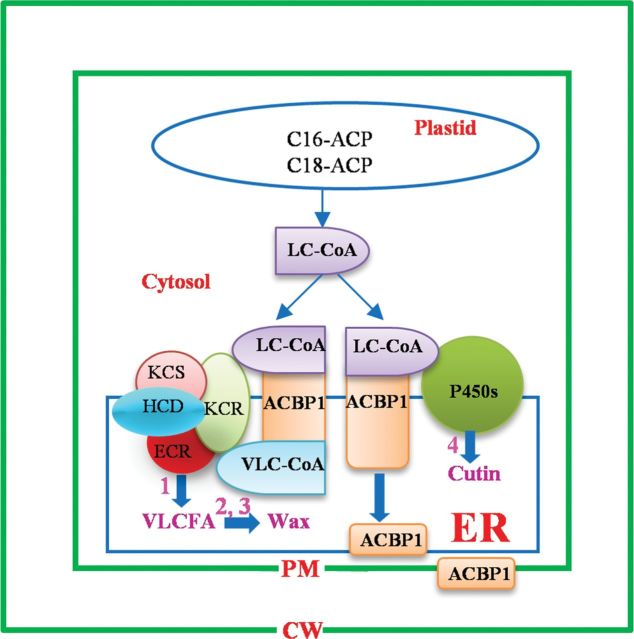
Proposed function of AtACBP1 in the wax and cutin synthesis pathway. The broad range of wax species altered in the *acbp1* mutant stem implies that the function of AtACBP1 in wax biosynthesis lies upstream in the pathway, involving VLCFA elongation (1). AtACBP1 may participate in general stem wax biosynthesis and could affect both the decarbonylation and acyl reduction pathways (2, 3). Changes in stem cutin monomer content in the *acbp1* mutant suggest that AtACBP1 participates in stem cutin synthesis (4). ACP, acyl carrier protein; LC-CoA, long-chain acyl-CoA; VLC-CoA, very-long-chain acyl-CoA; KCS, β-ketoacyl-CoA synthase; KCR, β-ketoacyl-CoA reductase; HCD, β-hydroxyacyl-CoA dehydratase; ECR, enoyl-CoA reductase; VLCFA, very-long-chain fatty acid; ER, endoplasmic reticulum; PM, plasma membrane; CW, cell wall.

In wax biosynthesis, C18 fatty acyl-CoAs are the predominant precursors for production of VLCFAs in four sequential reactions catalysed by a membrane-bound multiple enzyme system consisting of KCS, β-ketoacyl-CoA reductase (KCR), β-hydroxyacyl-CoA dehydratase (HCD), and enoyl-CoA reductase (ECR) (Kunst and Samuels, 2009). After several cycles of condensation of malonyl-CoA with long-chain acyl-CoAs, reduction to β-hydroxyacyl-CoA, dehydration to an enoyl-CoA, and reduction of the enoyl-CoA, VLCFAs with different acyl chains ranging from C20 to C34 are generated and subsequently converted to various wax components through decarbonylation and acyl reduction (Kunst and Samuels, 2003; Samuels *et al.*, 2008). Through multiple steps of hydroxylation and epoxidation, 16:0 and 18:X fatty acyl-CoAs are converted into cutin monomers (Schnurr *et al.*, 2004). ITC analysis from the present study revealed that rACBP1 binds not only long-chain acyl-CoA esters (C18:1-, C18:2-, and C18:3-CoA) but also saturated VLC acyl-CoA esters (C24:0-, C25:0-, and C26:0-CoA). The reduced binding affinity of rACBP1 for VLC acyl-CoA esters may be attributed to either the longer acyl chain length or unsaturation of the acyl chain. In measurements of rACBP1 interaction with VLC acyl-CoA esters, only C24:0 to C26:0 were tested because acyl-CoAs with acyl chains longer than C27:0 are not commercially available (http://www.avantilipids.com/). The present analysis demonstrated that AtACBP1 is able to bind C18 fatty acyl-CoAs and VLC acyl-CoAs, and can potentially transport these precursors for cutin and wax biosynthesis during stem cuticle formation (Fig. 8).

A reduction in wax crystal density and observations on the irregularity of the cuticle membrane on the stems of the *acbp1* mutant suggest that a defective cuticle had resulted from functional loss of AtACBP1. Interestingly, the T-DNA insertional mutants of *AtACBP3* also showed a highly irregular outermost cell wall surface (Xia *et al.*, 2012). These phenotypes are not only evident in *AtACBP* mutants but have also been observed in other mutants in cuticle development. Wax crystals were also absent on the stem surfaces of the *cer1*, *cer2*, and *cer6* mutants (Millar *et al.*, 1999). In addition, the *lacs2* mutant showed a reduction in plant size, seed set, and seedling establishment (Schnurr *et al.*, 2004). Furthermore, in the *wax2*, *fdh*, and *lcr* mutants, severe organ fusions occurred and pollen fertility was affected (Lolle *et al.*, 1998; Wellesen *et al.*, 2001; Chen *et al.*, 2003). These drastic phenotypic changes accompanied by severe reduction in wax or cutin load were caused by mutation in the genes of the wax and cutin pathways (Millar *et al.*, 1999; Wellesen *et al.*, 2001; Chen *et al.*, 2003; Lü *et al.*, 2009).

The wax species that were significantly lower in the *acbp1* mutant stem included not only alkanes, but also primary alcohols, C29 secondary alcohol, and ketones (Fig. 5A). This is in agreement with the down-regulation in the *acbp1* mutant stem of wax biosynthetic genes *CER8*, *KCR1*, *ECR*, and *CUT1/KCS6* (Fig. 6A), all of which are associated with VLCFA elongation. CER8 modiﬁes VLCFAs in both wax and cutin syntheses (Lü *et al.*, 2009); ECR (Zheng *et al.*, 2005) and KCR1 (Beaudoin *et al*., 2009) participate in VLCFA elongation; and CUT1/KCS6 is involved in the production of VLCFA precursors of stem wax (Millar *et al.*, 1999). The broad range of wax species altered in the *acbp1* mutant stem implied that the function of AtACBP1 in wax biosynthesis lies upstream in the pathway, involving VLCFA elongation (Fig. 8). It is proposed that AtACBP1 probably participates in general stem wax biosynthesis rather similar to the effect of CUT1/KCS6 in the pathway. Similarly, the decrease in cutin load in the stem of the *acbp1* mutant correlated well with the down-regulation of the cutin biosynthetic genes *LACS2*, *CYP86A2*, *CYP86A4*, and *GPAT8* (Fig. 6B). LACS2 is required for the correct assembly of the cuticular barrier (Schnurr *et al.*, 2004). CYP86A2 is a fatty acid ω-hydroxylase in the synthesis of hydroxy fatty acids (Xiao *et al.*, 2004), while CYP86A4 and GPAT8 catalyse ω-hydroxylation and esterification to glycerol, respectively, during cutin synthesis (Li *et al.*, 2007; Li-Beisson *et al.*, 2009). Knockout of *AtACBP1* probably adversely affected the accumulation of long-chain and VLC acyl-CoAs essential for stem wax and cutin biosyntheses (Fig. 8). Furthermore, the lack of substrates for wax and cutin biosyntheses led to a decrease in the expression of both stem wax and cutin biosynthesis genes, which will reduce wax or cutin production.

Waxes are known to be synthesized in the epidermis (Samuels *et al.*, 2008), and the leaf wax content was lower in the *acbp1* mutant in comparison with the wild type (Supplementary Fig. S2A at *JXB* online). However, *AtACBP1pro::GUS* was not observed to be expressed in the leaf epidermis (Fig. 2D), suggesting that AtACBP1 may not participate directly in leaf wax biosynthesis. Possibly, leaf wax changes may have been affected by the dramatic alterations observed in stem cuticular contents. Although the *AtACBP1pro::GUS*-transformed plants did not express detectable GUS activity in the leaf epidermal cells (Fig. 2C, D), reductions in several compounds (i.e. C31 and C33 alkanes, C28 fatty acid, and total wax load) in leaf wax but not leaf cutin (Supplementary Fig. S2) in the *acbp1* mutant may be attributed to a systemic change in the expression of wax-related genes and/or the activities of their gene products as a result of the defective stem cuticle. As the *acbp1* mutant exhibited lesions in stem cuticle formation, this could have potentially affected the status of the plant as a whole (e.g. water loss, susceptibility to pathogens, etc.), and could have culminated in an indirect effect on wax synthesis in the leaf epidermis. It is well documented that cuticular wax biosynthesis is sensitive to diverse environmental cues, and several transcription factors have been identified to play a role in its biosynthesis and accumulation (Aharoni *et al.*, 2004; Zhang *et al.*, 2007; Seo *et al.*, 2011; Cominelli *et al.*, 2008; Lü *et al*., 2009). Xia *et al.* (2012) showed that in the leaves of both *acbp3* and *acbp4* mutants, the cutin monomers were greatly reduced, with pronounced reduction in C16:0, C18:1, and C18:2 dicarboxylic fatty acids, but no change in most cutin monomers was evident in the *acbp6* mutant. In comparison, the present analysis revealed that stem cutin monomer levels also declined in the *acbp1* mutant, confirming its role in cutin biosynthesis (Fig. 8). In particular, C18 species (C18:1 and C18:2 ω-hydroxyl fatty acids and dicarboxylic fatty acids) of cutin were more affected in the *acbp1* mutant. This corresponds well to ITC data that showed that rACBP1 binds long-chain acyl-CoA esters (C18:1-, C18:2-, and C18:3-CoAs) with a greater affinity (i.e. smaller dissociation constant, *K*_D_) than VLC acyl-CoAs (C24:0-, C25:0-, and C26:0-CoA). AtACBP1 is localized in the PM and the ER, but AtACBP3 is targeted to the extracellular space, while AtACBP4 is a cytosolic protein. Although they show differential subcellular localization, they all affect cutin biosynthesis, suggesting that the binding and trafficking of precursors in cutin synthesis transverse across subcellular compartments.

The *acbp1* mutant in TEM showed an aberrant cuticle membrane in stems and was more susceptible to infection caused by *B. cinerea* possibly by entry through the aberrant cuticle, suggesting that alteration of cuticle constituents in this mutant impaired its basal defence responses. These results are consistent with the reduction in fungal pathogen resistance observed in the *acbp3*, *acbp4*, and *acbp6* mutants which were also cuticle-defective (Xia *et al.*, 2012). Previous findings have also revealed that AtACBP3 overexpression constitutively activated salicylic acid accumulation, *PR* gene expression and cell death, and increased resistance to the virulent bacterial pathogen *P. syringae* DC3000 (Xiao and Chye, 2011*b*).

Leaf susceptibility of the *acbp1* mutant to *B. cinerea* infection arising from a decline in leaf wax (but not leaf cutin) suggested that this decrease affected the leaf cuticle membrane through extrapolating the observations of altered cuticle in stems including significant decreases in both stem wax and cutin loads (Fig. 4). Lee *et al.* (2009*a*) reported that the *ltpg1* mutant showed a reduction in the C29 alkane in stems but not leaves, and they did not see any significant changes in total wax in neither stem nor leaf. Although *ltpg1* mutant leaves showed increases in three cutin constituents, they were more susceptive to *Alternaria brassicicola* (Lee *et al.*, 2009*a*). These findings support that changes in wax and cutin loads in stem and leaf may affect the cuticle barrier which is known to protect the plant against pathogen infection (Jenks *et al.*, 1994). Perhaps changes in cuticular content may also have altered the leaf surface structure which then enhanced susceptibility of the *acbp1* mutant to *B. cinerea*. Li *et al.* (2007) have reported that a change in the thickness or the structure of the pavement cells and the guard cells in the *gpat4gpat8* double mutant made it more susceptible to *A. brassicicola.* Similarly, in the *ltpg1* mutant which was more susceptible to *A. brassicicola*, Lee *et al.* (2009*a*) observed alterations in the structure of the cuticular layer, a protrusive cytoplasm, and disorganized grana and stroma lamellae in the chloroplasts.

In summary, using phenotypic and biochemical analyses of the *acbp1* mutant, it is demonstrated that AtACBP1 is involved in stem cuticle formation. Previous studies have suggested that plasma membrane-localized glycosylphosphatidylinositol-anchored lipid transfer proteins function in cuticular lipid transport (DeBono *et al.*, 2009; Lee *et al.*, 2009*a*; Kim *et al.*, 2012). It is illustrated herein that ER- and PM-associated AtACBP1 also participates in stem wax and cutin biosynthesis, probably as a carrier protein, as supported by ITC data (Fig. 1; Table 1).

## Supplementary data

Supplementary data are available at *JXB* online.

Figure S1. Expression analysis of wax and cutin biosynthetic genes, and genes with no implication on cuticle formation (triacylglycerol biosynthetic genes and cold-related genes) in stems of Col-0, the *acbp1* mutant, and the *acbp1-COM* line.

Figure S2. Cuticular wax and cutin monomer composition and amount in leaves of Col-0 and the *acbp1* mutant.

Table S1. Sequences of gene-specific primers for qRT-PCR.

Table S2. Mass-to-charge ratios (*m/z*) of cutin compounds used in mass spectrometry.

Supplementary Data

## Abbreviations:

ACBP: acyl-CoA-binding protein
ACP: acyl carrier protein
ECR: enoyl-CoA reductase
ER: endoplasmic reticulum
FID: flame ionization detector
GC: gas chromatography
GPAT: glycerol-3-phosphate acyltransferase
GUS: β-glucuronidase
HCD: β-hydroxyacyl-CoA dehydratase
ITC: isothermal titration calorimetry
KCR: β-ketoacyl-CoA reductase
KCS: β-ketoacyl-CoA synthase
LACS: long-chain acyl-CoA synthetase
MS: mass spectrometry
MS medium: Murashige and Skoog medium
PM: plasma membrane
TEM: transmission electron microscopy
SEM: scanning electron microscopy
VLCFA: very-long-chain fatty acid.
