# Supplementary material for: Arabidopsis membrane-associated acyl-CoA-binding protein ACBP1 is involved in stem cuticle formation
Source: J Exp Bot. 2014 Jul 22;65(18):5473–83. doi: 10.1093/jxb/eru304 (PMC4157719; doi:10.1093/jxb/eru304)
Supplement: Supplementary Data [file supp_65_18_5473__index.html]

 Arabidopsis membrane-associated acyl-CoA-binding protein ACBP1 is involved in stem cuticle formation — Supplementary Data 

# *Arabidopsis* membrane-associated acyl-CoA-binding protein ACBP1 is involved in stem cuticle formation

## Supplementary Data

Data files

**Files in this Data Supplement:**

- Supplementary Data - Supplementary Data
