# Supplementary material for: Arabidopsis membrane-associated acyl-CoA-binding protein ACBP1 is involved in stem cuticle formation
Source: J Exp Bot. 2014 Jul 22;65(18):5473–83. doi: 10.1093/jxb/eru304 (PMC4157719; doi:10.1093/jxb/eru304)
Supplement: Supplementary Data [file supp_eru304_Supplementary_data.pdf]

Supplementary data

Arabidopsis membrane-associated acyl-CoA-binding protein ACBP1 is involved in stem cuticle formation

Yan Xue, Shi Xiao, Juyoung Kim, Shiu-Cheung Lung, Liang Chen, Julian A. Tanner, Mi Chung Suh, Mee-Len Chye

Supplementary Figure S1.

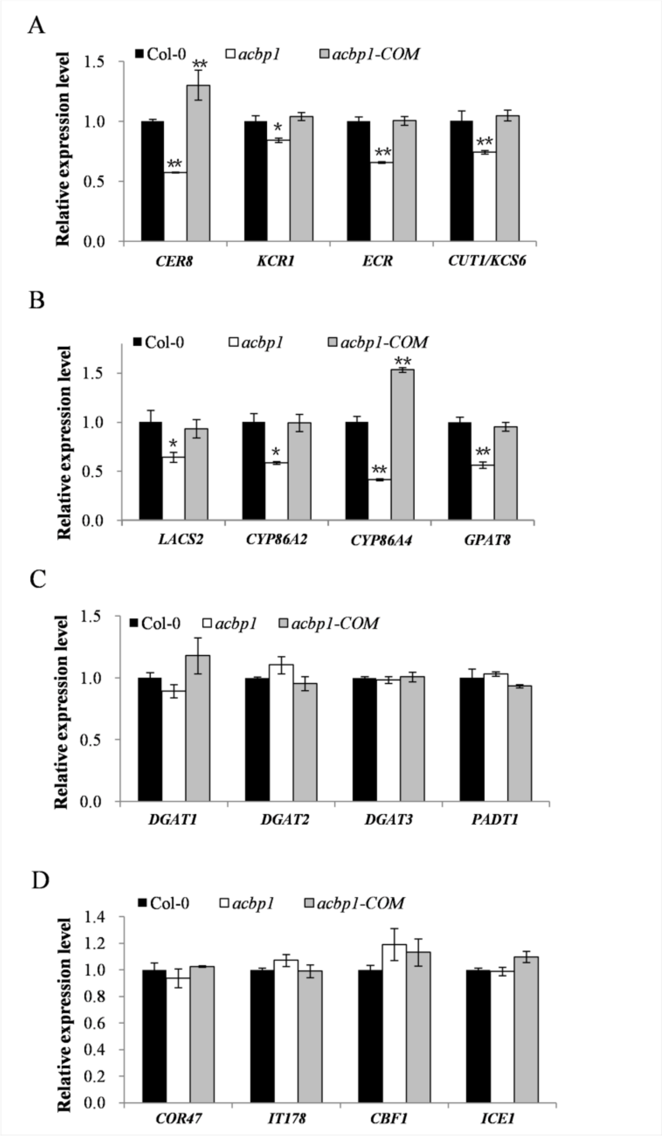

Figure S1. Expression analysis of wax biosynthetic genes (A), cutin biosynthetic genes (B), and genes with no implication on cuticle formation (triacylglycerol biosynthetic genes (C) and cold-related genes (D)) in stems of Col-0, the *acbp1* mutant and *acbp1-COM* line. Asterisks denote significant differences from the wild type (* $P < 0.05$, ** $P < 0.01$). Values are means ± SE (n = 3).

Supplementary Figure S2.

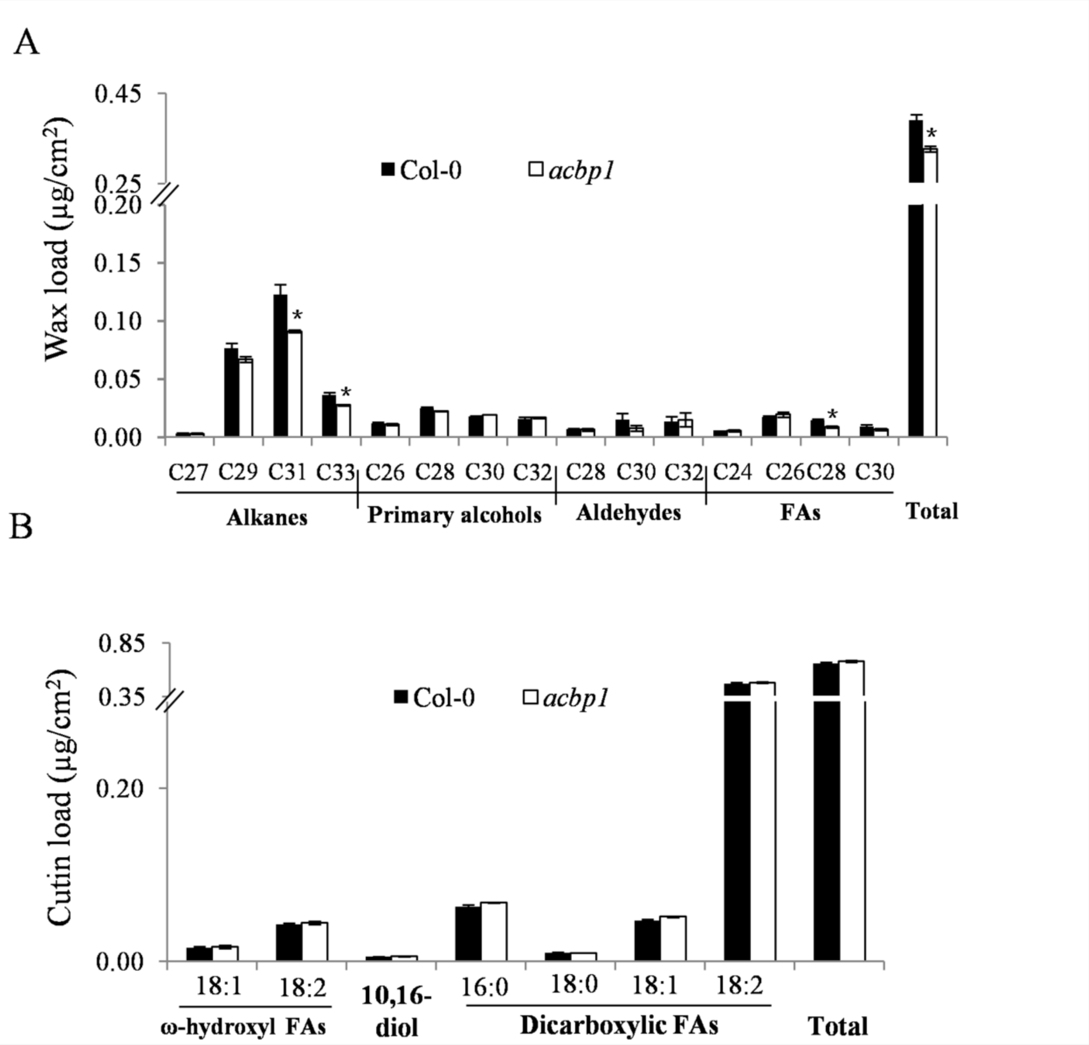

Figure S2. Cuticular wax (A) and cutin monomer (B) composition and amount in leaves of Col-0 and the *acbp1* mutant. Four-week-old leaves were used for wax and cutin analysis by gas chromatography (GC)-flame ionization detector and GC-mass spectrometry. FA, fatty acid; 10,16-diol, C16-10,16-dihydroxyl fatty acids. Asterisks denote significant differences from the wild type (* $P < 0.05$). Values are means ± SE (n = 3).

Supplementary Table S1. Sequences of gene-specific primers for qRT-PCR.

**Name**	**Sequence (5'→3')**	**Target gene**
ML1124	CCCGCTATGTATGTCGC	At3g18780 (*ACTIN2*)
ML1125	AAGGTCAAGACGGAGGAT	
ML1824	AGGCAAACATCACCACAATAGG	At1g68530 (*CUT1/KCS6*)
ML1825	CTCGTCGATCACCGCTCTG	
ML1826	AGCACCTTTGAGCCCTGAGA	At2g47240 (*CER8*)
ML1827	AACCGCTGGAATACCGACTG	
ML1828	ATTCCACGCGGTTTCCTCTTC	At3g55360 (*ECR*)
ML1829	GGCTTTCCATCTTTTCCATCAA	
ML1832	ACCCACTTGGCTCCTCATTCTC	At1g67730 (*KCR1*)
ML1833	GCTTTACCGATTCCGTCTGTTG	
ML1917	TAATACACGTAAGCAAGAAT	At4g00360 (*CYP86A2*)
ML1918	GTCGTGATGAGCCAGAAAAACC	
ML1919	GGCTCTTCCAACGCAAAACC	At1g01600 (*CYP86A4*)
ML1920	CGAACGCCAATCCGCAAATG	
ML1933	GATTGTGCCAGTAGCGATGAA	At4g00400 (*GPAT8*)
ML1934	TCTTGCCACCACCGTTGAC	
ML1937	AACGCAGCAATTTCGGTCCAG	At1g49430 (*LACS2*)
ML1938	AATGTCGCCACGCCAGTATCC	
ML2026	GGAGGGCGAGAGAGAGTCCACTT	At2g19450 (*DGAT1*)
ML2027	CCAATCTCGCAGCGATCTTGAAC	
ML2028	CCGAGCTGAGGAACATTCAAATCA	At3g51520 (*DGAT2*)
ML2029	CGACCATATTTGCTACGATGATCGA	
ML2030	GGTCTTGATCCTGAAGCTGGTTTG	At1g48300 (*DGAT3*)
ML2031	TCAGTCATCTTCTTCATGGCTTTGG	
ML2032	TGCTGAAGCAAAGGATGTTGCA	At5g13640 (*PDAT1*)
ML2033	CCTTTCTCCGGTGACCAATCAAG	
ML1716	GTCCACGCCGTTGGTTGT	At1g20440 (*COR47*)
ML1717	AATCCCCTTCTTCTCCTCCG	
ML1718	TTCCACCAGGGACAAAGG	At5g52310 (*LTI78*)
ML1719	CATCGTGTCCGTAAGAGGC	
ML1785	GGACTTTCCAAACCGCTGAG	At4g25490 (*CBF1*)
ML1786	CAAGCCGAGTCAGCGAAGTT	
ML1901	GATTTCTTGTCTGCTCGGTCACT	At3g26744 (*ICE1*)
ML1902	CCATTAGCAGGACTACCAAAACC	

The genes implicated in wax biosynthesis tested in qRT-PCR were *CUT1/KCS6*, *CER8*, *ECR* and *KCR1*. The genes implicated in cutin biosynthesis tested were

CYP86A2, *CYP86A4*, *GPAT8* and *LACS2*. The genes with no implication in cuticle formation tested included triacylglycerol biosynthetic genes (*DGAT1*, *DGAT2*, *DGAT3* and *PADT1*) and cold-related genes (*COR47*, *LTI78*, *CBF1* and *ICE1*).

Supplementary Table S2. Mass-to-charge ratios (m/z) of cutin compounds in mass spectrometry.

Cutin compound	Mass-to-charge ratios (m/z)
C17:0-FA (internal standard)	284
C15:0 cycloketone (internal standard)	282
C18:1-FA	296
C18:2-FA	294
C10,16-dihydroxyl fatty acids	343
C16:0-DCA	314
C18:0-DCA	342
C18:1- DCA	339
C18:2-DCA	337

FA, fatty acid; DCA, dicarboxylic fatty acid.
